# Distributed Storage Algorithm for Geospatial Image Data Based on Data Access Patterns

**DOI:** 10.1371/journal.pone.0133029

**Published:** 2015-07-16

**Authors:** Shaoming Pan, Yongkai Li, Zhengquan Xu, Yanwen Chong

**Affiliations:** 1 State Key Laboratory of Information Engineering in Surveying, Mapping and Remote Sensing, Wuhan University, Wuhan, Hubei, China; 2 Computer School of Wuhan University, Wuhan, Hubei, China; 3 Collaborative Innovation Center for Geospatial Technology, Wuhan, Hubei, China; University of Maribor, SLOVENIA

## Abstract

Declustering techniques are widely used in distributed environments to reduce query response time through parallel I/O by splitting large files into several small blocks and then distributing those blocks among multiple storage nodes. Unfortunately, however, many small geospatial image data files cannot be further split for distributed storage. In this paper, we propose a complete theoretical system for the distributed storage of small geospatial image data files based on mining the access patterns of geospatial image data using their historical access log information. First, an algorithm is developed to construct an access correlation matrix based on the analysis of the log information, which reveals the patterns of access to the geospatial image data. Then, a practical heuristic algorithm is developed to determine a reasonable solution based on the access correlation matrix. Finally, a number of comparative experiments are presented, demonstrating that our algorithm displays a higher total parallel access probability than those of other algorithms by approximately 10–15% and that the performance can be further improved by more than 20% by simultaneously applying a copy storage strategy. These experiments show that the algorithm can be applied in distributed environments to help realize parallel I/O and thereby improve system performance.

## Introduction

Declustering is one of the most effective methods in the field of parallel I/O and can be widely used to improve system performance by splitting and distributing large files among multiple storage nodes to speed up access to data. The Google file system (GFS) is a well-known distributed file system in which each large file is divided into several blocks of fixed size. Each block (approximately 64 megabytes (MB)) is then stored in multiple different storage nodes to enhance concurrency and system performance [1]. Moreover, a number of other similar systems, such as RAID (Redundant Array of Independent Disks) systems [2] and geospatial information systems (GISs) [3], have been developed, all of which use declustering technologies for the distributed storage of large files.

However, it is clearly imperative that we be able to store not only large files but also small files. With the rapid development of geospatial information technology and the widespread application of the Digital Earth system [4], an increasing number of small image files, most less than 64 MB in size, are being produced [5].

In fact, large amounts of small geospatial image files are currently stored in the Digital Earth system. Based on the multi-resolution pyramid approach to global satellite remote sensing images, remote sensing images are divided into image files of different resolution ratios, and each file is typically less than 64 MB. Examples of such systems include World Wind, Google Earth, Microsoft TerraServer [6], and the NASA Earth Observing System [7]. World Wind divides remote sensing images into small files, and these files are typically less than 1 MB in size [8,9]. Google Earth performs a similar type of processing; it splits files into slightly larger files, but the file sizes remain below 64 MB [10,11].

However, conventional declustering technologies, which play an important role in the field of distributed storage, still encounter difficulties in handling large numbers of small files [12], and further research on this issue is required [13]. To this end, a technology for the merging of small files has been proposed [14]. In the field of data storage, merging technologies are primarily used to reduce the numbers of files and the size of their metadata. HDWebGIS (WebGIS based on Hadoop) [15] is one typical example that is based on a proposed merging method that organizes and merges small files that are associated with similar spatial locations together into a single large file and then creates an index that is used to access the individual small files through middleware. Likewise, with the diffusion and application of cloud technology, the Hadoop distributed file system (HDFS), as one of the most prominent distributed file systems currently extant, must solve the problem of small file storage. Dong divides the small files that are stored on HDFS into three categories: structurally related, logically related and independent files [16]. Structurally related or logically related small files can be merged together and stored as a single large file to improve the performance of HDFS. Unfortunately, however, the cited study provides only a basic criterion for such merging; no specific method for merging small files based on their relationship is proposed.

Most previous studies have considered only the combination of small files into larger ones, followed by the distributed storage of each merged large file base on RAID technology. In fact, however, a particular block must be found and read from storage when a certain small file is requested, and this block cannot be prefetched when many requests for small files that belong to different merged files are issued simultaneously. Moreover, this process cannot be run in parallel, even when the small files are stored in the same storage node.

Given these challenges, this paper employs several strategies to organize and store small geospatial image data files into storage nodes in an attempt to optimize I/O parallelism performance in distributed environments. In this context, it is very important to understand, analyze and estimate the relationships among geospatial image data files that have a high probability of being requested simultaneously. To accomplish this goal, we analyze the data access patterns (**DAPs**) of geospatial image data files, which imply the relationships among these files, and then propose a new method of distribution on these DAPs to ensure that related small files (files with a higher probability of being requested simultaneously) are stored in different storage nodes to facilitate parallel requests.

## Overview of DAPs

DAPs are widely used in various fields for prefetching and caching [17]. James designed and implemented a Probability Graph (PG) to automatically predict future accesses based on DAPs, thereby greatly reducing the required cache size [18]. Thomas also proposed a Partitioned Context Modeling (PCM) approach, which was developed based on graph-based modeling, to improve the accuracy of predicting the next file to be accessed [19].

Hotmap is a typical DAP analysis system that is designed to analyze geospatial data access patterns (G-DAPs) based on the historical access log information produced by a GIS after a long period of operation (the server records the information related to geospatial image data files, such as the file name and image location, in chronological order when end users (clients) request image files from the server) [20]. According to the Hotmap model, a G-DAP satisfies Zipf’s law [21], which is described by Eq (1):

$$F_{i}=\theta /i^{\alpha }$$ (1)

where *F*_*i*_ is the number of accesses to the *i*th geospatial data file, *θ* is a constant and *α* is a parameter of Zipf’s law. Zipf’s law indicates which geospatial data files will be accessed more frequently. A number of advancements in caching and prefetching based on G-DAP utilization have been reported [22–24].

As mentioned by Thomas, DAPs can also be used to organize and adjust disk layouts [19]. Therefore, a new algorithm must be developed to solve the problem of small file storage in GIS applications.

## Distributed Storage of Geospatial Data Based on DAPs

By analogy with the Random distributed Storage Algorithm (**RSA**) and the Location-based distributed Storage Algorithm (**LSA**), which have been employed by several researchers [15,16], we refer to the algorithm proposed in this paper as the Access Pattern-based distributed Storage Algorithm (**APSA**). Table 1 summarizes the different storage strategies used in these algorithms.

**Table 1:** Storage strategies used by various algorithms.

Algorithms	Storage strategies
RSA	Randomly
LAS	Based on their locations
APSA	Based on their relationships

### 3.1 Description of APSA

We first provide some basic definitions of objects used by the algorithm.

First, let *F* = {*f*_1_, *f*_2_,…, *f*_*N*_} be the set of natural files, which includes all of the original small geospatial image data files (for brevity, we henceforth refer to small geospatial image data files simply as small files). Each element in *F* is labeled with a natural number [1, *N*], and *N* is the total number of small files. The natural numbers [1, *N*] can then be defined as a natural file vector *I*_*o*_ = (1,2,⋯, *N*) based on the natural sequence of these files.

Let *C* = {*c*_1_, *c*_2_,…, *c*_*m*_} denote the set of storage nodes, where *m* is the total number of all storage nodes. For simplicity, each of the *N* small files is stored in *m* storage nodes on average (an uneven grouping can be transformed into an even grouping by copying select small files that have higher request rates; this process is demonstrated in section 4, and a related experiment is detailed in section 5.4).

Finally, let $\tilde{F}=\{{\tilde{F}}_{1},{\tilde{F}}_{2},\ldots ,{\tilde{F}}_{m}\}$ be the set of grouped storage files. Each group of small files will be stored in one of the storage nodes, and each small file in *F* will belong to one and only one group. In other words, the element ${\tilde{F}}_{i}=\{{\tilde{f}}_{i1},{\tilde{f}}_{i2},\ldots ,{\tilde{f}}_{in}\}$ in $\tilde{F}$ is a group of *n* small files that will be stored in the *i*th storage node, *c*_*i*_. Here, *m* × *n* = *N*, and *n* is called the storage length.

Furthermore, for $\forall {\tilde{F}}_{i}\in \tilde{F}$ (*i* ∈ [1, *m*]), if we assume that the small files ${\tilde{f}}_{i1},{\tilde{f}}_{i2},\ldots ,{\tilde{f}}_{in}$ are labeled by *t*_*i*1_, *t*_*i*2_,⋯, *t*_*in*_ in *F* and are denoted by *T*_*i*_ = (*t*_*i*1_, *t*_*i*2_,⋯, *t*_*in*_) (*t*_*ij*_ ∈ [1, *N*], *i* ∈ [1, *m*], *j* ∈ [1, *n*]), then *T* = (*T*_1_, *T*_2_, ⋯, *T*_*m*_)^*T*^ = (*t*_*ij*_)_*m*×*n*_ defines the map from set *F* to set $\tilde{F}$, and each *T*_*i*_ is a restriction of *T* on $\left\{f_{t_{i1}},f_{t_{i2}},\ldots ,f_{t_{in}}\right\}$. Therefore, the map $T:F\Rightarrow \tilde{F}$ denotes a storage distribution rule that defines how the small files are assigned to storage nodes on average. If the storage distribution vector is denoted by *I*=(*t*_11_,*t*_12_,⋯,*t*_1*n*_,*t*_21_,*t*_22_,⋯,*t*_2*n*_,⋯,*t*_*m*1_,*t*_*m*2_,⋯,*t*_*mn*_), then the **APSA** key can be converted to construct an *N* × *N* permutation matrix, *B*, that satisfies the condition *I* = *I*_*o*_ *B*. We can then achieve our goal of distributing all small files across all storage nodes on average.

### 3.2 Access correlation matrix

To construct *B* (or to find an optimal *T*), we must analyze the historical access log information [20], which reflects the small files’ access patterns and can be used to compute the relationships among the small files.

Let $R=\{f_{a_{1}},f_{a_{2}},\ldots ,f_{a_{M}}\}$ denote the chronological access sequence of small files that is obtained from the historical access log information recorded by the server of the Digital Earth system. The vector *A* = (*a*_1_, *a*_2_,⋯, *a*_*M*_) can then be defined as the geospatial data file access vector. Here, *M* is the total number of accesses to all small files in *F*, and the natural number *a*_*i*_ ∈ [1, *N*] (*i* = 1,2,⋯, *M*) denotes the label of the *i*th requested file from *F* (i.e., *a*_*i*_ = *k* (*i* = 1,…, *M*), which indicates that the *i*th requested file is *f*_*k*_ (*k* ∈ [1, *N*])).

Because the storage length is *n*, we divide *A* into several *n*-element sub-vectors; then, *A* can be written as *A* = (*S*_1_, *S*_2_, ⋯, *S*_*l*_), where *S*_*i*_ = (*a*_*i*1_, *a*_*i*2_, ⋯, *a*_*in*_) (*a*_*ij*_ ∈ [1, *N*], 1 ≤ *i* ≤ *l*, 1 ≤ *j* ≤ *n*) is an *n*-element sub-vector in *A* and *l* is the total number of *n*-element sub-vectors. The set of all sub-vectors of vector length *n* is denoted by *S* = {*S*_*k*_: *k* ∈ [1, *l*]}. For ∀*S*_*k*_ ∈ *S*, let ${\tilde{S}}_{k}=\{a_{k1},a_{k2},\cdots ,a_{kn}\}$ denote the set of all *n* elements of sub-vector *S*_*k*_. We can then define the access correlation function for all small files as shown in Eq (2); this function represents the relationship between any pair of small files:

$$R_{S_{k}}(i,j)=\left\{ \begin{array}{l} 1\;i\in {\tilde{S}}_{k},\;j\in {\tilde{S}}_{k},\;i\neq j\\ 0\;Others \end{array}\right.\;1\leq i\leq N,\;1\leq j\leq N,\;1\leq k\leq l$$ (2)

Here, $R_{S_{k}}(i,j)$ denotes the access correlation between *f*_*i*_ and *f*_*j*_ during a short period of access time. Therefore, $R_{S_{k}}(i,j)$ indicates whether the geospatial data files *f*_*i*_ and *f*_*j*_ are both likely to be requested within a short period of time. If $R_{S_{k}}(i,j)$ = 1, then we consider that *f*_*i*_ and *f*_*j*_ have one storage conflict, and we define the following:

$$R_{S}(i,j)=\sum_{k=1}^{l}R_{S_{k}}(i,j)\;1\leq i\leq N,\;1\leq j\leq N$$ (3)

*R*_*S*_(*i*, *j*) represents the total number of storage conflicts between *f*_*i*_ and *f*_*j*_. A larger value of *R*_*S*_(*i*, *j*) indicates a higher level of storage conflict or a higher total concurrent access probability **(TCAP)** between the small files *f*_*i*_ and *f*_*j*_, corresponding to a higher probability that these files will be assigned to different storage nodes to achieve a higher total parallel access probability **(TPAP)** in the case that they are requested simultaneously.

For all small files in *F*, we can obtain an *N* × *N* matrix based on *S* as follows:

$$R_{S}={\left(R_{S}(i,j)\right)}_{N\times N}\;1\leq i\leq N,\;1\leq j\leq N$$ (4)

Here, *R*_*S*_ is called the access correlation matrix and represents the concurrent access correlations among the small files. This matrix has the following properties (as proven in the appendices):

1. *R*_*S*_ is a symmetric matrix, i.e., *R*_*S*_^*T*^ = *R*_*S*_;
2. $\sum_{i=1}^{N}\sum_{j=1}^{N}R_{S}(i,j)=(n-1)M\equiv K_{S}$;
3. $P(f_{i})≅\sum_{j=1}^{N}R_{S}(i,j)/\sum_{i=1}^{N}\sum_{j=1}^{N}R_{S}(i,j)=\sum_{j=1}^{N}R_{S}(i,j)/{\text{K}}_{\text{S}}\;i\in [1,N]$; and
4. $P_{S}(f_{j}|f_{i})≅R_{S}(i,j)/\;\sum_{j=1}^{N}R_{S}(i,j)=K_{S}R_{S}(i,j)/P(f_{i})\;i,j\in [1,N]$.

Here, *P*(*f*_*i*_) is the concurrent access probability of *f*_*i*_ in *S*, and *P*_*S*_(*f*_*j*_|*f*_*i*_) is the conditional probability that *f*_*j*_ will belong to the same *S* to which *f*_*i*_ belongs.

### 3.3 Mathematical model of APSA

Let *T*_*i*_ = (*t*_*i*1_, *t*_*i*2_, ⋯, *t*_*in*_) be an arbitrary row vector in *T*. Then, according to *T*_*i*_, we can store *n* geospatial data files ${\tilde{F}}_{i}=f_{\;T_{i}}=\{f_{t_{i1}},f_{t_{i2}},\ldots ,\;f_{t_{in}}\}$ in the *i*th storage node *c*_*i*_. If any file in $f_{T_{i}}$ is requested, then $f_{T_{i}}$ is requested (in other words, the storage node *c*_*i*_ is busy and is unable to serve any other clients), and $P(f_{T_{i}})$ denotes the concurrent access probability of the set of small files $f_{T_{i}}$. On the basis of the 3rd property of *R*_*S*_ stated in section 3.2, we can define the following:

$$P(f_{T_{i}})=\sum_{j=1}^{n}P(f_{t_{ij}})=\sum_{j=1}^{n}(\sum_{k=1}^{N}R_{S}(t_{ij},k)/{\text{K}}_{\text{S}}\;)=\frac{1}{K_{S}}\sum_{j=1}^{n}\sum_{k=1}^{N}R_{S}(t_{ij},k)\;i\in [1,m]$$ (5)

Similarly, if $H(f_{T_{i}})$ denotes the conditional probability of the set of small files $f_{T_{i}}$ (representing the probability that if any file in $f_{T_{i}}$ is requested, the other files in $f_{T_{i}}$ will be requested within a short period of time), then on the basis of the 4th property of *R*_*S*_ stated in section 3.2, we can define the following:

$$\begin{array}{l} H(f_{T_{i}})=\sum_{j=1}^{n}\left[P(f_{t_{ij}}|f_{T_{i}})\sum_{p=1}^{n}P(f_{t_{ip}}|f_{t_{ij}})\right]\\ \;=\sum_{j=1}^{n}[\frac{\sum_{k=1}^{N}R_{S}(t_{ij},k)}{\sum_{j=1}^{n}\sum_{k=1}^{N}R_{S}(t_{ij},k)}\frac{\sum_{p=1}^{n}R_{S}(t_{ij},t_{ip})}{\sum_{k=1}^{N}R_{S}(t_{ij},k)}]=\frac{\sum_{j=1}^{n}\sum_{p=1}^{n}R_{S}(t_{ij},t_{ip})}{\sum_{j=1}^{n}\sum_{k=1}^{N}R_{S}(t_{ij},k)}\;i\in [1,m] \end{array}$$ (6)

Eqs (5) and (6) define the relationship among *R*_*S*_, the access patterns and the concurrent access probabilities of the small files. For simplicity, let $P(R_{S},T_{i})=\sum_{j=1}^{n}\sum_{k=1}^{N}R_{S}(t_{ij},k)$ and $\text{H}(R_{S},T_{i})=\sum_{j=1}^{n}\sum_{p=1}^{n}R_{S}(t_{ij},t_{ip})$; then, Eqs (5) and (6) can be rewritten as follows:

$$P(f_{T_{i}})=P(R_{S},T_{i})/K_{S}$$ (7)

$$H(f_{T_{i}})=H(R_{S},T_{i})/P(R_{S},T_{i})$$ (8)

Furthermore, according to the definition of the map $T:F\Rightarrow \tilde{F}$, the **TCAP** $H(\tilde{F})$ can be defined as in Eq (9):

$$H(\tilde{F})=\sum_{i=1}^{m}P({\tilde{F}}_{i})H({\tilde{F}}_{i})=\sum_{i=1}^{m}P(f_{T_{i}})H(f_{T_{i}})$$ (9)

By combining (7), (8) and (9), we obtain the following:

$$H(\tilde{F})=\sum_{i=1}^{m}\frac{P(R_{S},T_{i})}{K_{S}}\frac{H(R_{S},T_{i})}{P(R_{S},T_{i})}=\frac{1}{K_{S}}\sum_{i=1}^{m}H(R_{S},T_{i})=\frac{1}{K_{S}}H(R_{S},T\;)$$ (10)

where

$$H(R_{S},T)=\sum_{i=1}^{m}H(R_{S},T_{i})=\sum_{i=1}^{m}\sum_{j=1}^{n}\sum_{p=1}^{n}R_{S}(t_{ij},t_{ip})$$ (11)

Eq (10) describes the explicit relationship between the objective function $H(\tilde{F})$ and both *R*_*S*_ and *T*. As mentioned above, the goal of **APSA** is to achieve parallel access to geospatial data files, which requires a **TPAP** that is as high as possible. Therefore, the goal of **APSA** can be restated as the attempt to obtain the lowest possible **TCAP**, and we can conclude that $H(\tilde{F})$ is proportional to *H*(*R*_*S*_, *T*). Therefore, the mathematical model of **APSA** can be defined as follows:

$$T^{*}=\underset{T}{\text{arg min}}(H(R_{S},T))$$ (12)

If we obtain an optimal *T* according to Eq (12), then we also obtain an optimal *B*. However, this is a typical NP-hard problem, and the traversal search method is impractical because of the extremely large amount of calculation time required. Therefore, the goal of the algorithm must be modified to obtain a reasonable solution. This modification is discussed in the next section.

## Practical Heuristic Algorithm for APSA

According to the APSA description given in section 3, the orders in and among the groups of $\tilde{F}$ are meaningless, and thus, the underlying problem is typical of unordered average combinations. Therefore, we can develop a heuristic algorithm to obtain an optimal *T* using Eq (12). This process includes 3 main steps: 1) obtain *F* from the historical access log information, 2) generate *R*_*S*_ using the algorithm proposed in section 3.2 and reorder *R*_*S*_ to reduce the scale of the search; and 3) employ a locally approaching search method to find the optimal *T*.

### 4.1 Preprocessing of *F*

Large numbers of small files are stored in the Digital Earth system, and therefore, we must process a large collection of natural files. For example, for the 90-meter-resolution global terrain data files from the Shuttle Radar Topography Mission (SRTM90), the length of *I*_*o*_ will be 3,538,890 and the size of *R*_*S*_ will be 3538890×3538890. However, as indicated by the geospatial DAP, only approximately 20% of these files will be requested [21–23]. Therefore, we only need to process a subset of the data, which allows us to reduce the size of *R*_*S*_.

To satisfy the requirements for storing all *N* small files in *m* storage nodes on average, we can copy certain geospatial data files that have higher request rates and assign new labels to the copies (to expand the scale of *F* and *I*_*o*_).

### 4.2 Preprocessing and reordering of *R*_*S*_

The historical access log information is produced by the Digital Earth system after a long period of operation, and from this information, we can obtain *A*. Then, we can generate *R*_*S*_ using the algorithm described in section 3.2.

To reduce the search scale, we must concentrate non-related elements together to allow the storage distribution rule *T* to be rapidly sought and obtained. Thus, we can reorder *R*_*S*_ using the RCM ordering algorithm, which was developed based on the CM ordering algorithm [25–26]. We can then obtain a new *P*, in which most of the nonzero elements are concentrated along the diagonal.

The objective of **APSA** is to generate grouped geospatial data files and to ensure that these groups have the smallest possible storage conflict, meaning that the value of *R*_*S*_ is zero or near zero. To employ the RCM ordering algorithm to concentrate the non-related elements along the diagonal, we must first preprocess *R*_*S*_ in two steps: 1) denote the largest value of *R*_*S*_ by *R*_max_ and search for and obtain *R*_max_ from *R*_*S*_, and 2) ∀*R*_*S*_(*i*, *j*), set *R*_*S*_(*i*, *j*) = *R*_max_ – *R*_*S*_(*i*, *j*) (*i* < *j* ≤ *N*).

Afterward, we can use the RCM algorithm to reorder *R*_*S*_, export the resulting permutation *P* in reverse order and then export the corresponding matrix *P*_*S*_ (the standard RCM algorithm is used in this paper; therefore, a description of this process is omitted).

### 4.3 Determination of the optimal *T* using a locally approaching search algorithm

Several steps are required to obtain the optimal *T*:

1. Initialize *T* = (0)_*m*×*n*_ and set *k* = 1.
2. Let *i* be the label of the first row in *P*_*S*_; then, a non-zero length can be obtained: *non_zero_len* = max{|*i* − *j*|, *A*_*ij*_ ≠ 0}.
3. If *non_zero_len* ≤ *n*, then for every *j* ∈ [1, *n*], let *T*[*k*][*j*] = *P(j)*. Then, delete the *n* top rows of *P*_*S*_ and delete the first *n* elements of *P*. Go to 7).
4. If *non_zero_len* > *n*, then take the upper triangular matrix *UTM*_*m*_ = {*A*_ij_, 1 ≤ *i* ≤ *j* ≤ *non_zero_len*}. Let *X* = (*x*_1_, *x*_2_,⋯⋯, *x*_*n*_) denote an *n*-dimensional temporary vector and initialize *x*_1_ = 1, *i*_1_ = 1, and *j* = 2. Set the basis vector of the local search to *B* = *UTM*_*m*_(*i*_1_).
5. While *j* ≤ *n*, search for the largest element *B*(*i*_*j*_) in *B*. Then, the label of the *j*th file is *i*_*j*_; set *x*_*j*_ = *i*_*j*_. Update the basis vector of the local search as follows: *B* = *B* + *UTM*_*m*_(*i*_*j*_). Set *B*(*i*_*j*_) = -*K*_*S*_ and *j* = *j* + 1.
6. For 1 ≤ *j* ≤ *n*, set *T*[*k*][*j*] = *P(x*_*j*_ *)*. Then, delete the *n* rows of *P*_*S*_ that are defined in the temporary vector *X* and delete the corresponding *n* elements of *P*.
7. Set *k* = *k* + 1. If *k* ≤ *m*, then return to 2); otherwise, stop.

For the optimal *T*, *n* small files are included in each group, i.e., the *i*th group includes small files labeled as *T*[*i*][1], *T*[*i*][2], $#x2026;$#x2026;, *T*[*i*][*n*], and the relationship among the files in a given group is as weak as possible. Therefore, the *i*th group of files can be stored in the *i*th storage node *c*_*i*_.

## Experimental Results and Analysis

To evaluate the performance of the algorithm, several tasks were experimentally investigated: 1) selecting the geospatial image dataset to be stored in distributed storage nodes; 2) finding the optimal *T* based on the historical access log information recorded by the Digital Earth server [20] using the heuristic algorithm proposed in section 4; 3) requesting the same dataset simultaneously based on other historical access log information; and 4) computing the **TPAP** performance and comparing it with those of LSA and RSA.

We define the **TPAP** performance as follows:

$$TPAP=\frac{\sum_{i=1}^{L}\sum_{j=1}^{m}x_{ij}}{L\times m}$$ (13)

where *L*×*m* denotes the total number of requests for small files over a long period and *x*_*ij*_ denotes whether the *j*th storage node is accessed during a short period. Specifically, ∀*i* ∈ [1, *L*], if *c*_*j*_ is accessed during this short period, then *x*_*ij*_ = 1; otherwise, *x*_*ij*_ = 0. Therefore, the value of *TPAP* cannot exceed 1.

The simulation algorithm was implemented using Microsoft Visual C++6.0, and the sequences were accessed and processed using MATLAB R2009a (Version 7.8.0.347) in accordance with the rules specified in section 4. All datasets are summarized in Table 2. Two types of datasets were included: geospatial image datasets produced by our own simulation system [27] and an INS (Instructional Workload) dataset obtained from the University of California at Berkeley [28].

**Table 2:** The datasets used in this analysis.

Category	Dataset	Number of access sequences	Dataset size^a^	Access sequence length^b^
1	SRTM30	5	10,000	180,000~204,000^*^
2	SRTM30	10	2,000~10,000	180,000~204,000^*^
3	SRTM90	2	10,000	200,000^*^
4	Landsat7	2	10,000	200,000^*^
5	INS	3	20,000	244,339~712,605^**^

^a^All data were relabeled with natural numbers ranging from zero to the length of the dataset.

^b^Each access sequence recorded only the labels of the data in chronological order.

^*^ As stated in section 4.1, only of the 20% files will be requested.

^**^ All files will be requested.

### 5.1 Contrasting experiments on different algorithms

**Fig 1:**
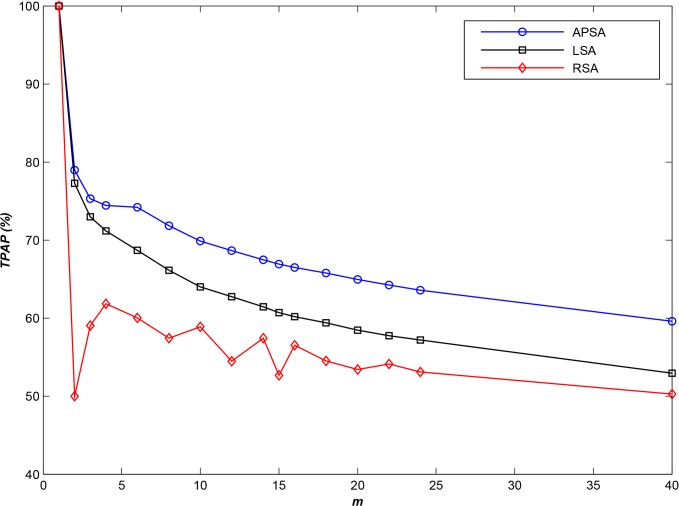
Comparison of *TPAP* results at various scales of *m* (*N* = 10,000).

From the 30-m-resolution global terrain data files from the Shuttle Radar Topography Mission (SRTM30), we selected **10,000** geospatial image data files from a given spatial region for use as the experimental dataset. All available sequences of access log information for the considered SRTM30 datasets are summarized in Table 2 (category 1 and category 2). We determined the optimal *T* using the first access log information file in category 1, and we then assessed the performance of the various algorithms using the second log file in category 1. Fig 1 presents the results of the comparison at different scales of *m*.

As shown in Fig 1, APSA and LSA exhibit comparable performance in a small-scale environment, especially when *m* is less than 8. In this case, only a small number of storage nodes are available, and most of the small files must be stored in the same storage node. Moreover, the majority of clients will request small files according to their navigation paths [22–24], and therefore, LSA, which stores small files in different storage nodes depending on their spatial locations, can satisfy the requirements of parallel I/O.

For a larger number of storage nodes, however, **APSA** performs better than **RSA** or **LSA**, especially when *m* is larger than 22. The performance of **APSA** is higher than that of **LSA** by approximately 10% and higher than that of **RSA** by approximately 15%, especially for a large number of servers.

**Fig 2:**
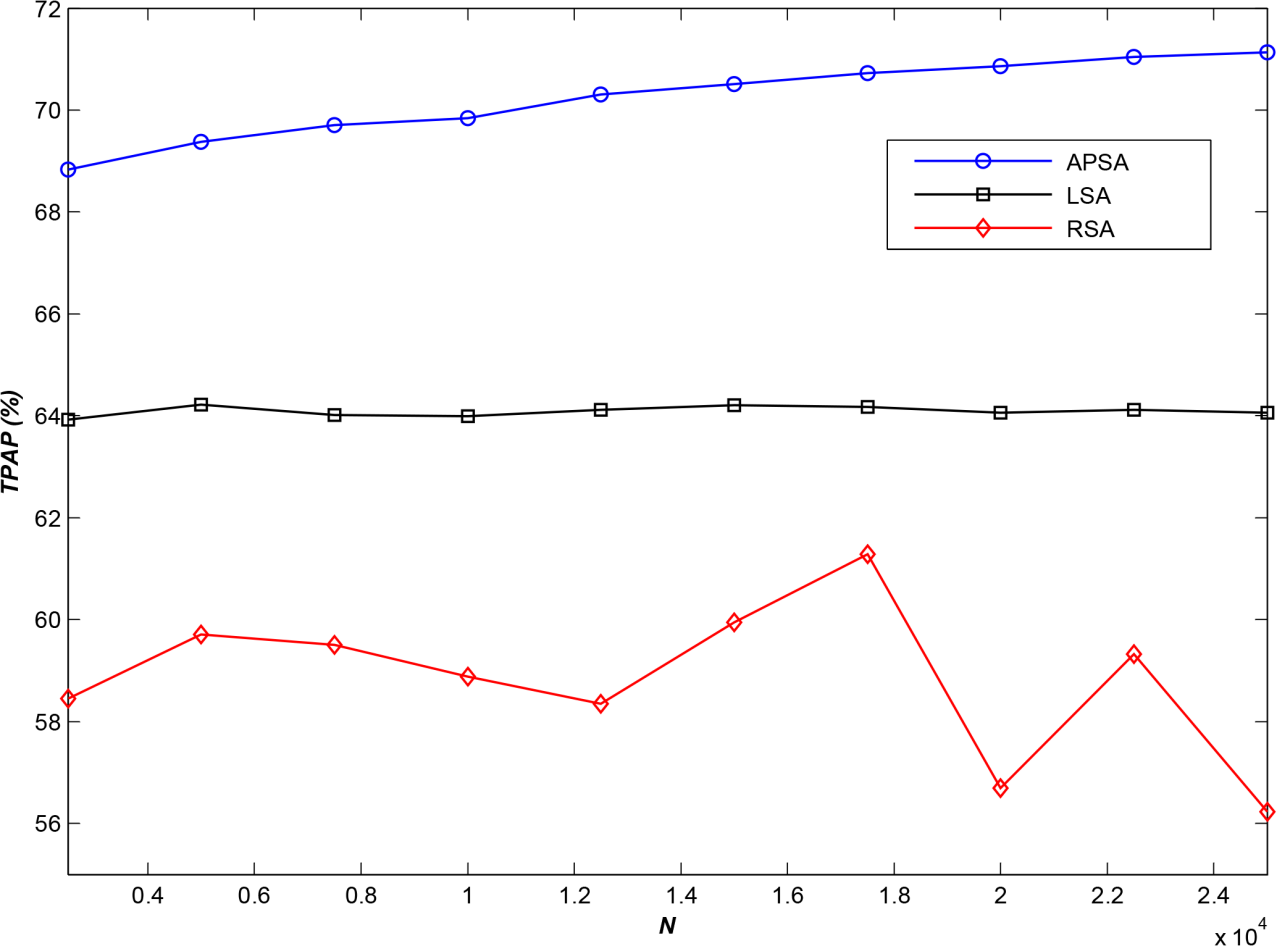
Comparison of *TPAP* results for various values of *N* (*m* = 10).

Furthermore, we tested the performance of APSA using all log files in category 2, which represented various scales of small files. Fig 2 displays the results of the comparison for various values of *N*.

As shown in Fig 2, as the scale of the geospatial data increases, the performance of LSA exhibits almost no change, and the performance of RSA becomes considerably more unstable and fragmented and even decreases to some extent. By contrast, the performance of **APSA** improves, exhibiting a sustained increase. The experimental results show that the proposed algorithm offers greater advantages in a large-scale environment (i.e., for a large number of storage nodes).

### 5.2 Experiments using different types of access log information

**Fig 3:**
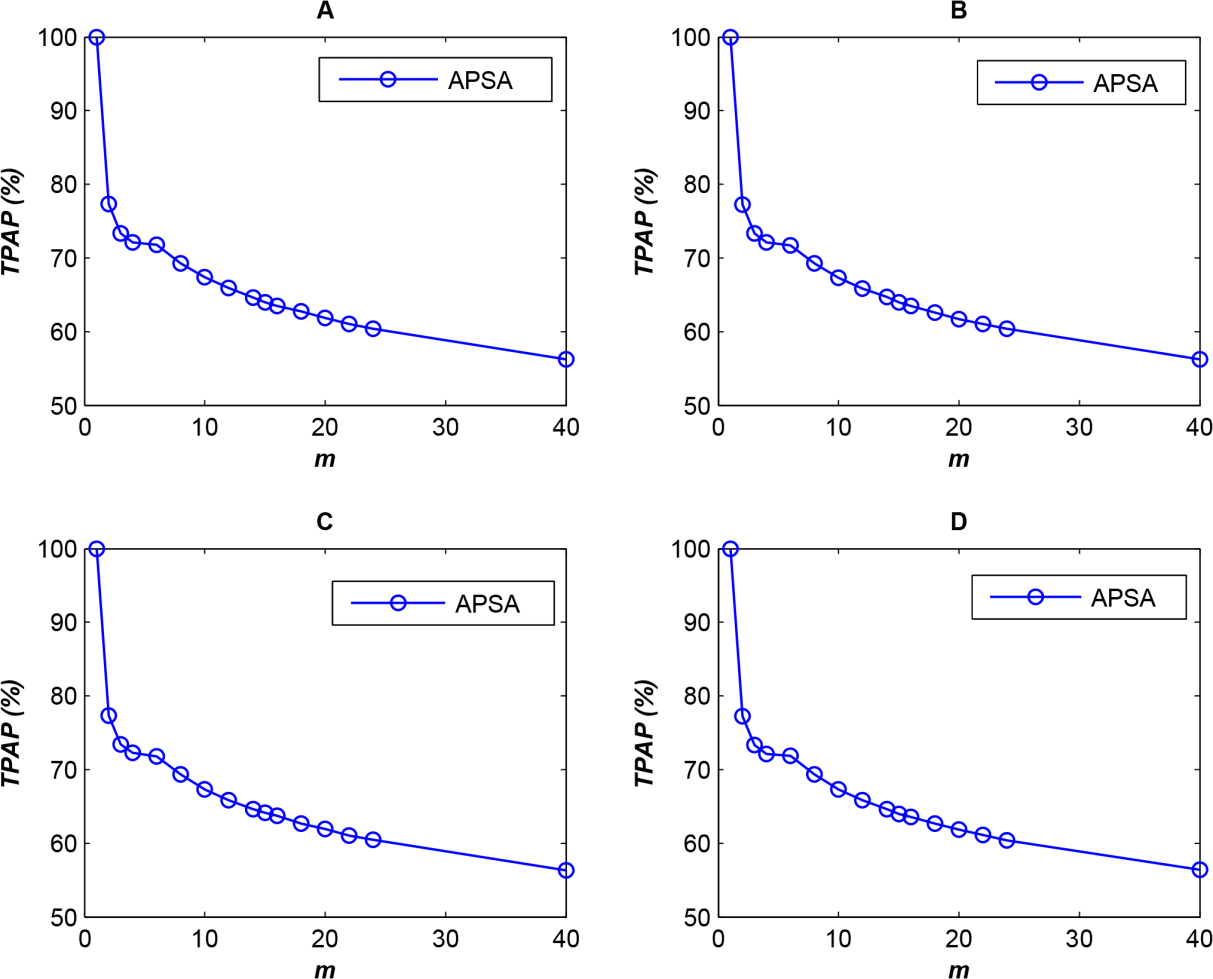
Comparison of *TPAP* results for different access log files (*N* = 10,000) (each plot, A-D, represents a different access log file).

It is important to determine whether an algorithm can always provide high performance. To assess the adaptability of the algorithm, we selected four typical access log information files representing the access behavior of end users (clients) at different times. As in the first experiment, we used the first log file from category 1 to determine the optimal *T* and then used the 2nd through 5th log files of category 1 to test the performance of APSA. The experimental results are shown in Fig 3A–3D).

As shown in Fig 3, comparable performances were obtained when different access log files were used to obtain a feasible solution. In addition, the rate of change in ***TPAP*** did not exceed **6%**. Thus, this experiment demonstrated that **APSA** exhibits stable parallel access performance under different conditions.

### 5.3 Experiments using different geospatial image datasets

To assess the adaptability of the algorithm to different geospatial image datasets, we selected three typical geospatial datasets: SRTM30, SRTM90 and Landsat7 ETM+[29]. In this experiment, the first two log files from categories 1, 3 and 4 were used.

**Fig 4:**
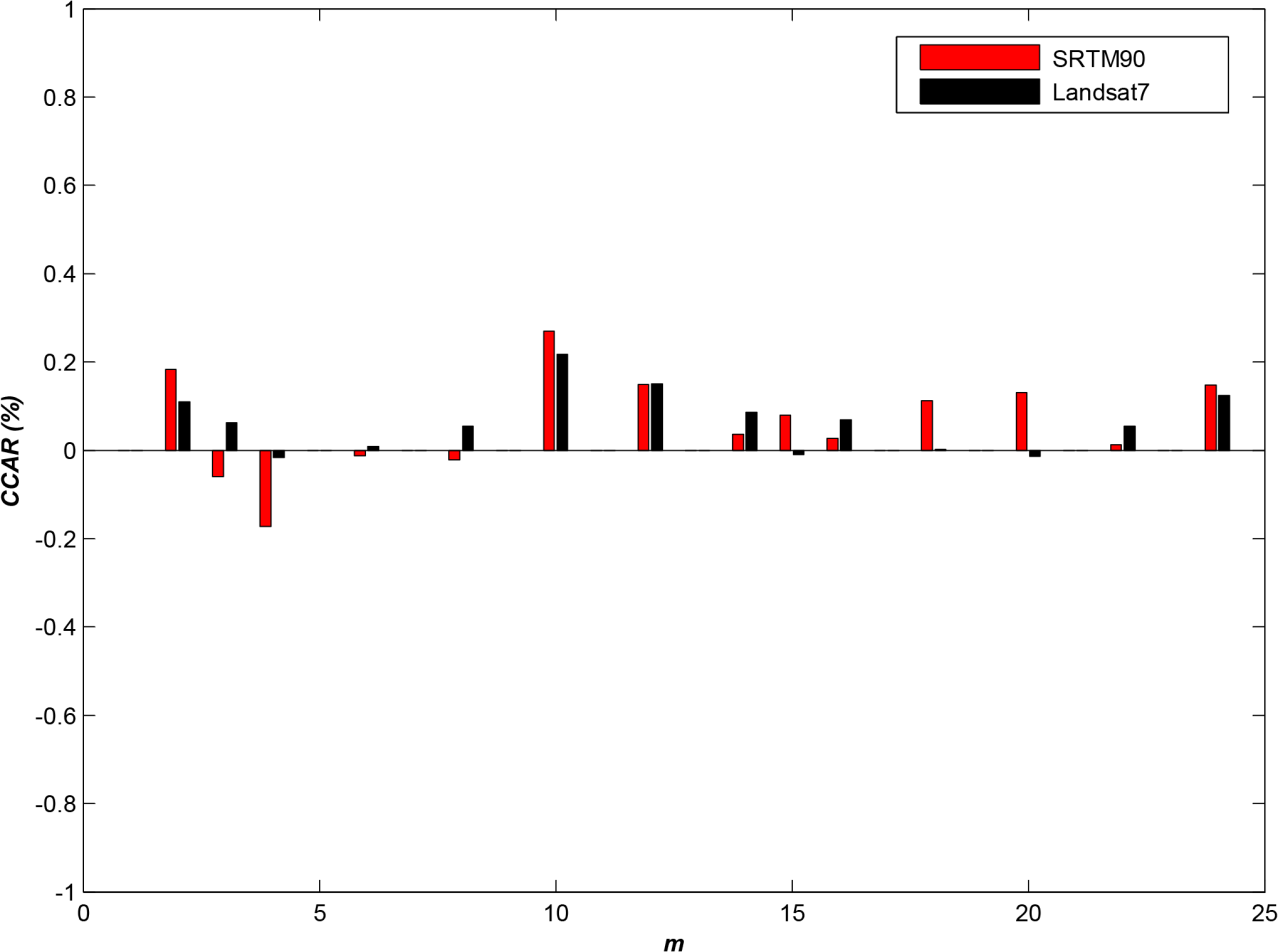
The adaptability of APSA to different datasets (*N* = 10,000).

Let *TPAP*_*SRTM* 30_ be the performance indicator for SRTM30, let *TPAP*_*SRTM* 90_ be the performance indicator for SRTM90, and let *TPAP*_Landsat7_ be the performance indicator for Landsat7. Then, the ***TPAP*** change rate (*CCAR*) can be calculated using Eq (14). The experimental results are shown in Fig 4.

{CCARSRTM90=(TPAPSRTM90−TPAPSRTM30)TPAPSRTM30CCARLandsat7=(TPAPLandsat7−TPAPSRTM30)TPAPSRTM30 (14)

As shown in Fig 4, ***CCAR*** exhibits essentially no change, and the highest rate of change does not exceed **0.4%** and thus is generally negligible. Therefore, the experiment shows that **APSA** demonstrates broad adaptability to different geospatial datasets.

### 5.4 Experiments based on redundant data storage

To simplify data processing and enhance the efficiency of the algorithm, we assume (as noted in the **Appendix** (**Property 2**)) that any given small file will not be repeatedly requested within a short period of time. Nevertheless, in actuality, certain small files do exist that are repeatedly requested within short periods of time.

However, most storage solutions adopt a copy storage strategy for data security; examples of such systems include RAID, which copies and stores each datum to backup disks, and RADOS (Reliable, Autonomic Distributed Object Store), which manages a number of copies on demand [30].

**Fig 5:**
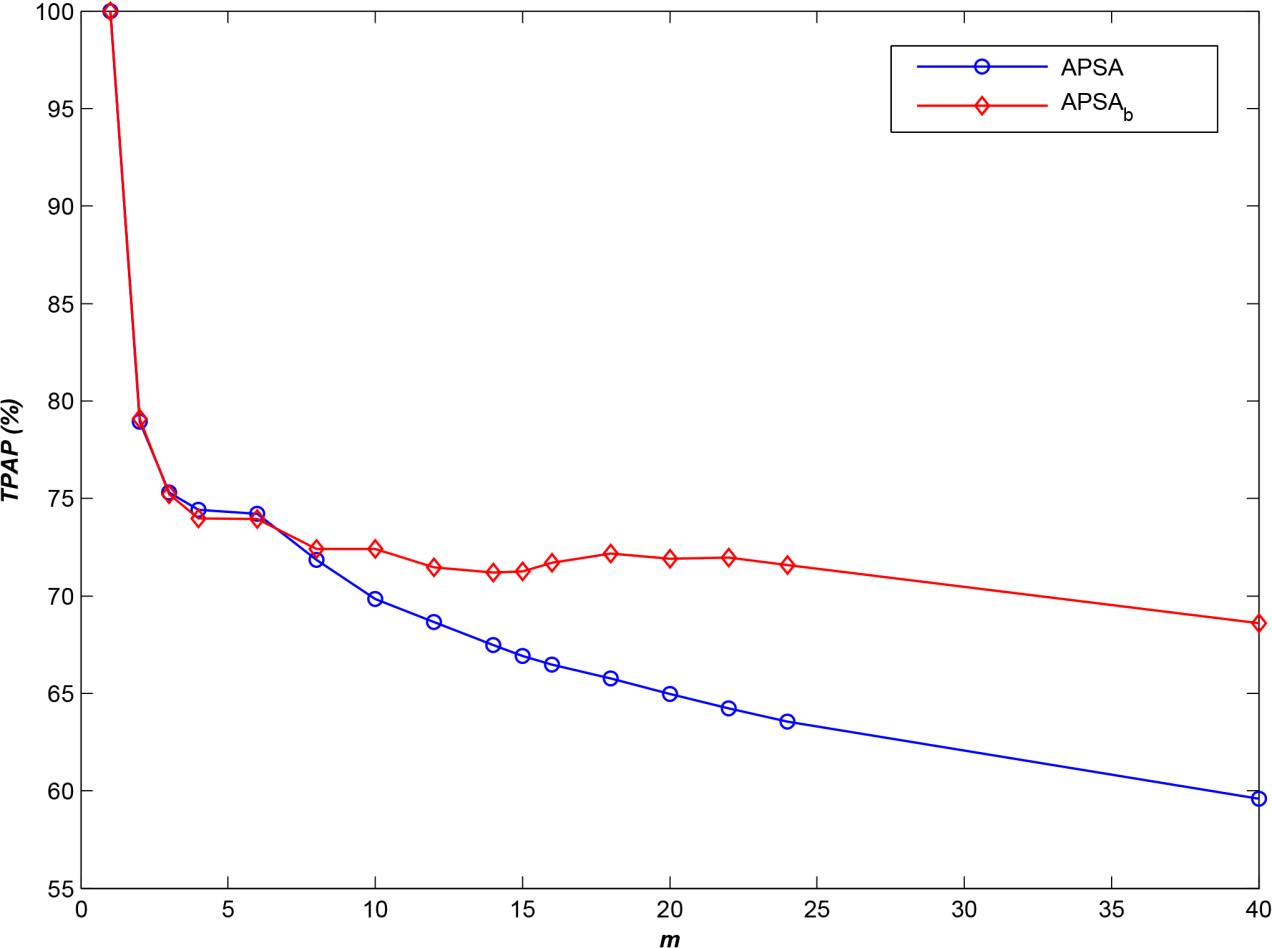
The performance improvement achieved using the copy storage strategy (*N* = 10,000, *CR* = 6.3%).

**Fig 6:**
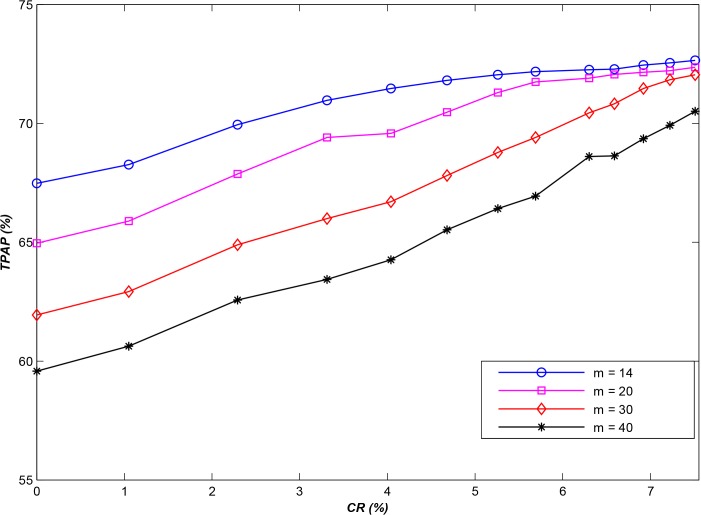
The performance improvement achieved using the copy storage strategy with various *CR*s (*N* = 10,000).

Inspired by this approach, we can copy select geospatial data files that have higher request rates, and then we can store them in different storage nodes with new labels. Let *CR* be the ratio of the number of copies to the total number of geospatial data files; next, a larger *CR* obviously implies the generation of more copies. Based on the results of the first experiment, Fig 5 displays a comparison between the original APSA and **APSA**_**b**_, for which *CR* is 6.3% (the geospatial data files with the highest access rates are each copied only once). Furthermore, comparisons between the original APSA and variants of **APSA**_**b**_ with different *CR*s are shown in Fig 6.

As shown in Fig 5, the performance of **APSA**_**b**_ is essentially identical to that of APSA for small numbers of storage nodes, especially when *m* is less than 8. In this case, only a small number of clients can access the system simultaneously (for simplicity, we assume that one storage node can provide service to only one client at a time; in fact, all clients share the resources of the storage node, including CPUs and bandwidth, when they simultaneously request service from the same storage node, but the server may require the same amount of time to serve all end users simultaneously as it does to serve each client sequentially). Therefore, there is only a very small probability that more than one client will attempt to access the same geospatial data file simultaneously, and therefore, the copy storage strategy will not function effectively. However, as the scale of the system increases, the performance improves considerably, especially for an *m* greater than 22; indeed, the performance can be improved by more than **20%**.

Obviously, larger *CR*s mean more copies, and more copies result in a higher probability that different clients can simultaneously access the same geospatial data files in parallel. Fig 6 shows the corresponding improvement in performance as *CR* increases. However, achieving continuous improvement in the system performance is difficult once *CR* reaches a certain size, because some of the small files will be simultaneously accessed at certain times and accessed in a staggered manner at others. This situation cannot be fully resolved using a copy storage strategy alone, and it can only be partially resolved through algorithm optimization to obtain a better solution than that identified by the method introduced in section 4. Such algorithm optimization will be a focus of our future studies.

### 5.5 Adaptability experiments based on another type of dataset

In this section, we consider another typical type of log file, namely, INS (Instructional Workload) files, to test the adaptability and flexibility of our solution. The INS files used in this experiment were obtained from separate distributed file system application environments at the University of California at Berkeley. Roselli [28] traced two groups of Hewlett-Packard series 700 workstations running HP-UX 9.05. INS data were collected from twenty machines in these groups, which were located in laboratories for undergraduate classes.

**Fig 7:**
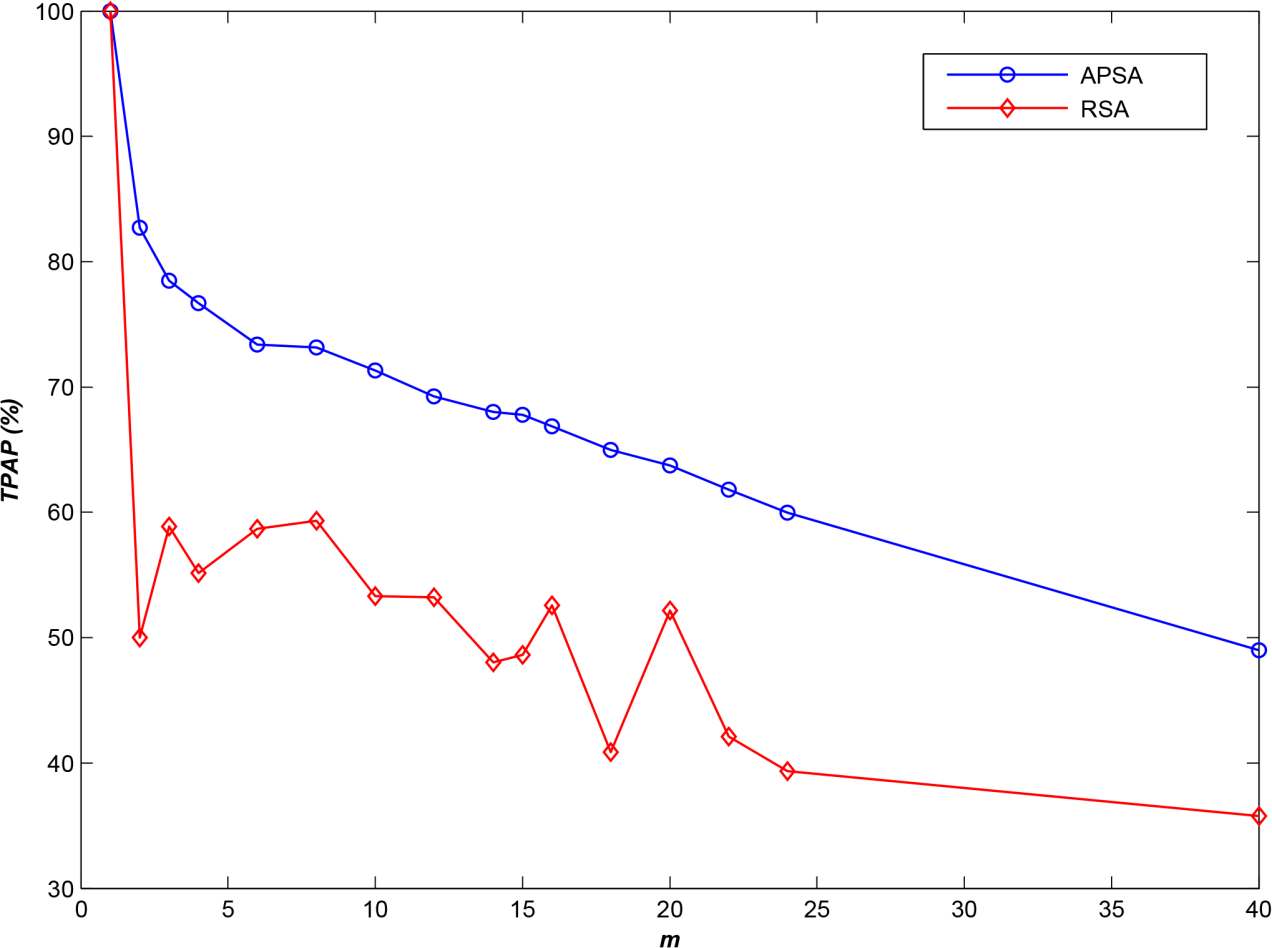
Adaptability experiment based on the INS dataset (*N* = 20,000).

As in the experiments presented in section 5.1, we selected **20,000** files for use as the experimental datasets and employed different access log information files to obtain the optimal *T* and to simulate file access. Thus, we were able to compute *TPAP* at various storage node scales. All log files for the INS dataset are summarized in Table 2 (category 5). Fig 7 presents the results of a comparison between APSA and RSA on these files (LSA was not used because there was no location relationship among the INS files).

**Fig 8:**
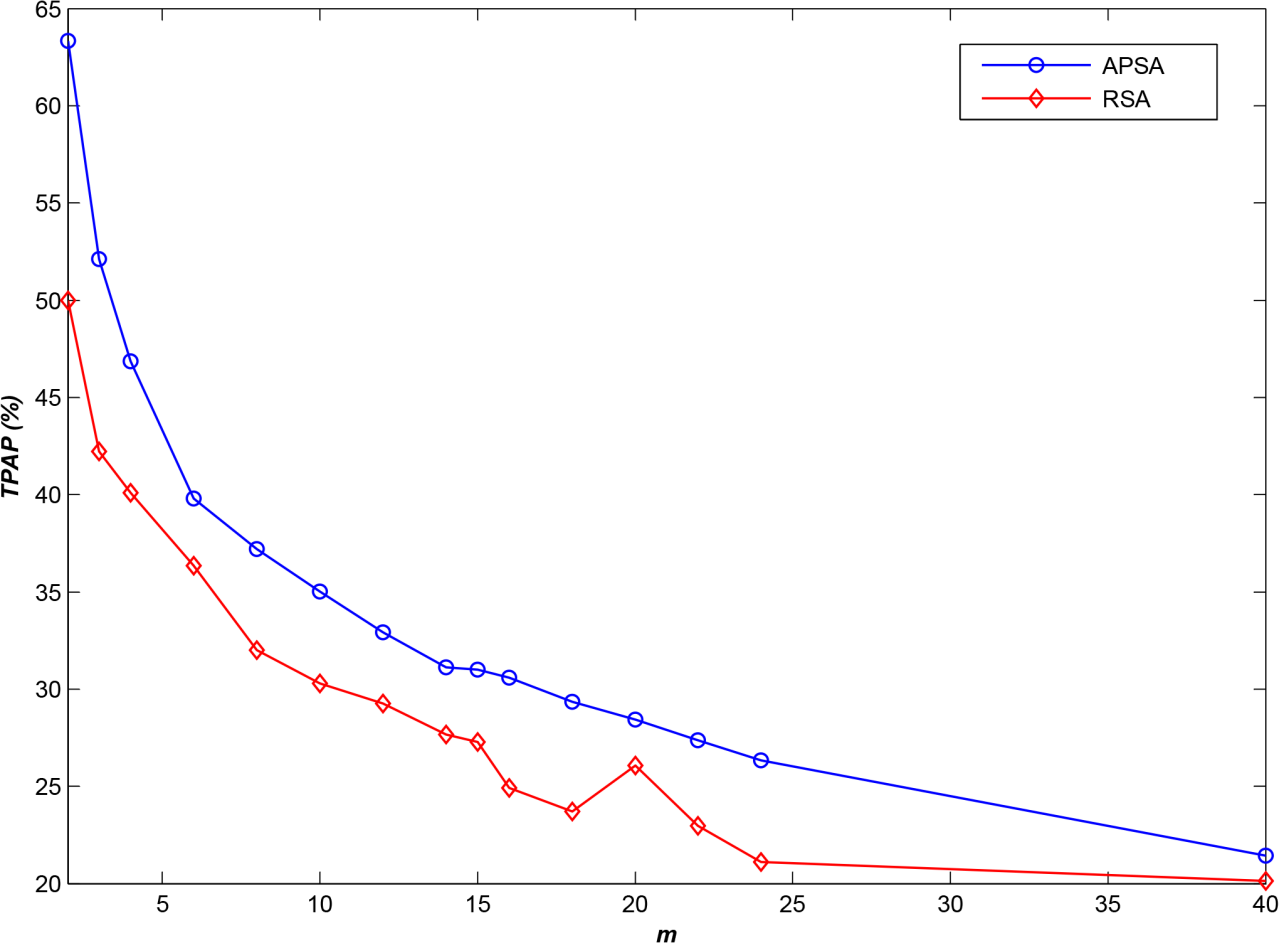
Experimental results for another access log file (*N* = 20,000).

**Fig 9:**
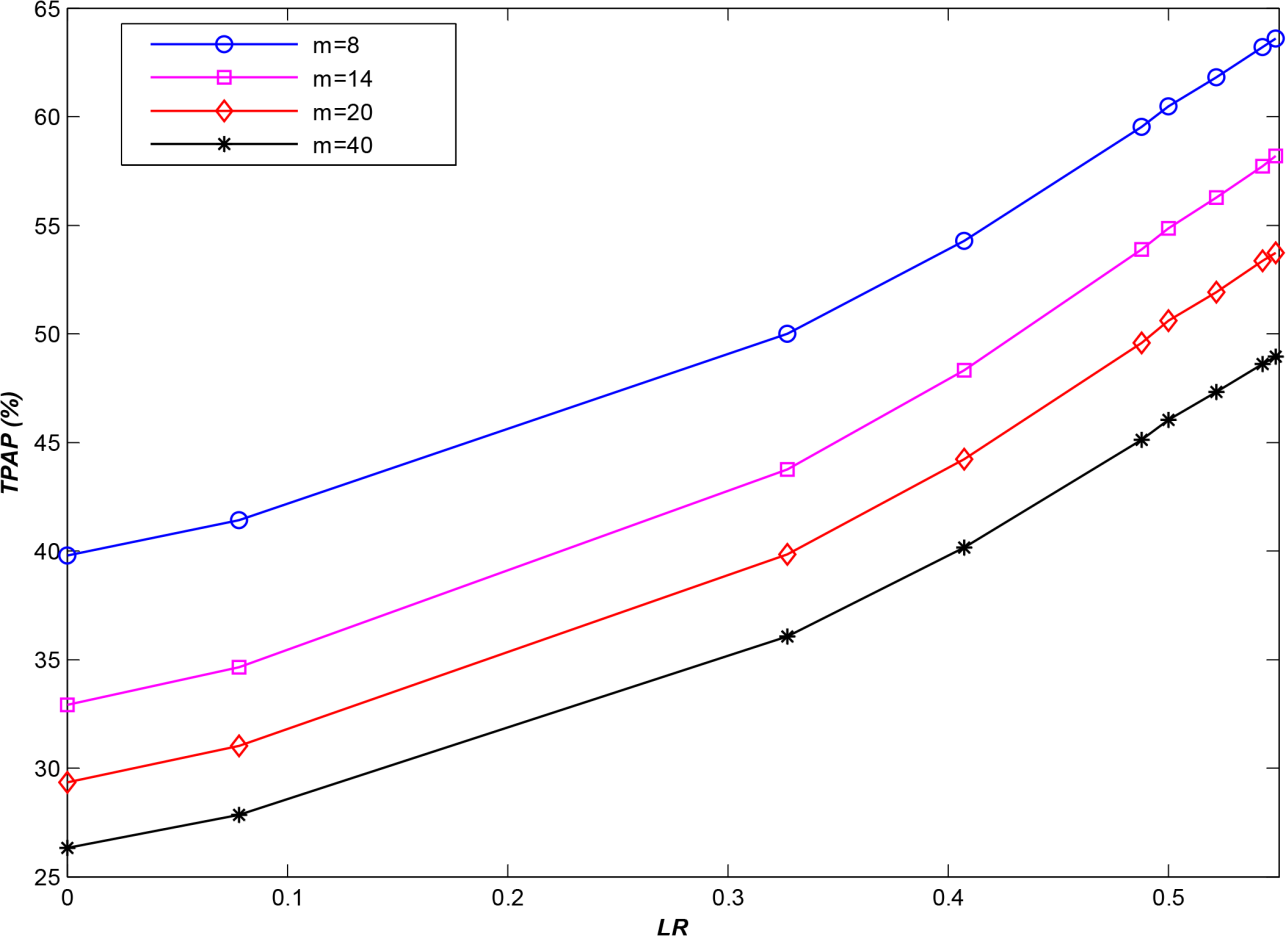
The performance improvement at various *LR*s (*N* = 20,000).

As shown in Fig 7, APSA demonstrates higher performance than does RSA (by approximately 18%) when applied to this type of general dataset. However, INS datasets include not only small files but also several large files. Therefore, when end users (clients) access these large files, they will occupy storage node resources for a longer time, and therefore, the performance of **APSA** will decline sharply (as shown in Fig 8). Nevertheless, the performance of **APSA** is still higher than that of **RSA**, by approximately 10%. Furthermore, if we ignore accesses to large files, where *LR* is the ratio between the minimum length of the ignored sequences and the total sequence length, then the results are as shown in Fig 9 for various *LR*s. From this figure, it is evident that the performance of **APSA** can be effectively improved by increasing *LR*. Recall that, as mentioned in section 1, declustering technologies can be used to satisfy the requirements of parallel I/O in distributed environments; therefore, hybrid storage strategies are required to satisfy the requirements for both small and large files. The development of such a hybrid strategy will be a focus of our future investigations.

## Conclusions

In this paper, we investigated the challenges associated with the distributed storage of small files and proposed a complete theoretical system for distributing small geospatial data files for storage. First, we discussed the patterns of access to geospatial data files and created a theoretical mathematical model to express the relationship among geospatial data files. Then, we developed a practical heuristic algorithm to find an acceptable optimization solution.

To verify the developed mathematical model, a series of comparative experiments was performed, as described in section 5. All of these comparative experiments demonstrate that our method can achieve a higher ***TPAP*** than other algorithms can (by approximately **10–15%**) and that its performance can be further improved by more than **20%** using a copy storage strategy. Most importantly, all experiments show that APSA can satisfy the requirements for the storage of a large amount of small files in a distributed environment.

Although our algorithm exhibits higher performance than any of the other tested strategies, it is based solely on historical access log information; there is no provision for dynamically updating the strategies, even when the hotspots change (i.e., when the relationship among the small files changes) [31]. Thus, the establishment of a rapid and efficient updating mechanism that considers and tracks such changes in hotspots is worth further investigation. Moreover, hybrid storage strategies that satisfy the requirements for the storage of both large and small files should also be further investigated.

However, our paper only considers how small geospatial image data files can be separately stored into different storage nodes to improve system I/O performance. The nodes also include many very tiny files, some of which must be merged together to avoid storage fragments. One such example is sport activity files, which include a significant amount of information such as GPS locations, distance, speed, calories, and heart-rate; these files are widely used to optimize athletes’ training [32]. Due to differences in duration and GPS distance, the sizes of sport activity files will vary substantially, as some may only require a few kilobytes (KB), while others may reach a size of 10 MB [33]. Thus, a type of integrated storage strategy that can satisfy the requirements of very tiny files, small files and large files should also be considered.

## Appendix: Proof of the Properties of *R*_*S*_

### Property 1

From Eq (3), it is clear that $R_{S_{k}}(i,j)=R_{S_{k}}(j,i)$. Then, we can obtain the following:

$$R_{S}(i,j)=\sum_{k=1}^{l}R_{S_{k}}(i,j)=\sum_{k=1}^{l}R_{S_{k}}(j,i)=R_{S}(j,i)$$ (A-1)

Therefore, *R*_*S*_(*i*, *j*) = *R*_*S*_(*j*, *i*); that is, *R*_*S*_^*T*^ = *R*_*S*_, indicating that *R*_*S*_ is a symmetric matrix.

### Property 2

Several studies have shown that a chronological access sequence of small files, *R*, follows a Markov process; such processes are widely used in predictive models [21, 34–35]. In accordance with this model, *A* = (*a*_1_, *a*_2_,⋯, *a*_*M*_) can be treated as a Markov chain with a state space *I*_0_ = [1, *N*], where the number of states is *N*. Moreover, based on the G-DAPs, we can assume that the service system has been running for a sufficiently long time before we obtain its access log information *R* that the distribution of the small files in *R* is stationary. Therefore, *A* can be regarded as a stationary Markov chain.

Let Π = {*π*_*i*_: *i* ∈ *I*_0_} denote the stationary distribution of the pattern of access to *F* = {*f*_1_, *f*_2_,…, *f*_*N*_}, let *P* = (*p*_*ij*_)_*N*×*N*_ denote the transition matrix, and let *p*_*ij*_ = *P*(*a*_*k*+1_ = *j*|*a*_*k*_ = *i*) (*i*, *j* ∈ *I*_0_, *k* ∈ [1, *M*]) be the (*i*,*j*)th element of *P*. We then have Π = ΠP. Thus, ∀*j* ∈ *I*_0_, we have $\pi_{j}=\sum_{i=1}^{N}\pi_{i}p_{ij}$, and *π*_*i*_ exhibits no relationship with its location. Therefore, for any set $\tilde{S}$ of all sub-vectors *S*_*k*_, ∀*i* ∈ *I*_0_, the probability that the *i*th state appears in $\tilde{S}$ is as follows:

$$P(i\in \tilde{S})=1-{\left(1-\pi_{i}\right)}^{n}$$ (A-2)

Because of the large number of small files stored in the system, *N* >> 1, and thus, *π*_*i*_ << 1. Therefore, we find that (1 − *π*_*i*_)^*n*^ ≈ 1 – *nπ*_*i*_, and thus, Eq (A-2) can be rewritten as follows:

$$P(i\in \tilde{S})≅n\pi_{i}$$ (A-3)

Let $P^{\left(1\right)}(i\in \tilde{S})$ denote the probability that the *i*th state appears in $\tilde{S}$ only once; then, $P^{\left(1\right)}(i\in \tilde{S})$ can be defined as in Eq (A–4):

$$P^{\left(1\right)}(i\in \tilde{S})=C_{n}^{1}\pi_{i}{\left(1-\pi_{i}\right)}^{n-1}\approx n\pi_{i}-n(n-1)\pi_{i}^{2}$$ (A-4)

If $P^{\left(>1\right)}(i\in \tilde{S})$ denotes the probability that the *i*th state occurs in $\tilde{S}$ more than once, then we obtain the following:

$$P^{\left(>1\right)}(i\in \tilde{S})=P(i\in \tilde{S})-P^{\left(1\right)}(i\in \tilde{S})$$ (A-5)

By combining (A-2), (A-3) and (A-4), we find that

$$P^{\left(>1\right)}(i\in \tilde{S})=n(n-1)\pi_{i}^{2}$$ (A-6)

Based on the above analysis, *π*_*i*_ << 1, and thus, $P^{\left(>1\right)}(i\in \tilde{S})$ is negligible. Therefore, the one broad conclusion that can be drawn from this result is that all elements of $\tilde{S}$ are different; this conclusion is consistent with actual observations of G-DAPs, which indicate that a given small file will not be requested repeatedly within a short period of time. We can then write the following:

$$\sum_{i=1}^{N}\sum_{j=1}^{N}R_{\tilde{S}}(i,j)=\sum_{i,j\in S_{k}}R_{\tilde{S}}(i,j)=2C_{n}^{2}=n(n-1)$$ (A-7)

Therefore,

$$\begin{array}{l} \sum_{i=1}^{N}\sum_{j=1}^{N}R_{S}(i,j)=\sum_{i=1}^{N}\sum_{j=1}^{N}\sum_{k=1}^{l}R_{S_{k}}(i,j)=\sum_{k=1}^{l}\sum_{i=1}^{N}\sum_{j=1}^{N}R_{S_{k}}(i,j)\\ \;=\sum_{k=1}^{l}n(n-1)=n(n-1)l=(n-1)M \end{array}$$ (A-8)

Eq (A–8) shows that the sum of all elements in *R*_*S*_ is a constant that is equal to (*n*-1)*M*. Therefore, if we let *K*_*S*_ = (*n* − 1)*M*, then we can write the following:

$$\sum_{i=1}^{N}\sum_{j=1}^{N}R_{S}(i,j)=(n-1)M\equiv K_{S}$$ (A-9)

### Property 3

According to the analysis presented for **Property 2** and Eq (A–3), ∀*i* ∈ [1, *N*] and *k* ∈ [1, *l*], we can write the following:

$$P(f_{i})=\pi_{i}=\frac{1}{n}\underset{S_{k}\in S}{P}(i\in S_{k})$$ (A-10)

Let ${\tilde{S}}_{i}=\{S_{k}:k\in [1,l],\;i\in S_{k}\}$ and let $C_{i}=card({\tilde{S}}_{i})$; then, the frequency of $S_{k}\in {\tilde{S}}_{i}$ in *S* is as follows:

$$\underset{S_{k}\in S}{\bar{P}}(S_{\;k}\in {\tilde{S}}_{i})=C_{i}/l$$ (A-11)

Note that $\sum_{i\in S_{k},j=1}^{N}R_{S_{k}}(i,j)=n-1$ and $\sum_{i\notin S_{k},\;j=1}^{N}R_{S_{k}}(i,j)=0$. Then, we can write the following:

$$\begin{array}{l} \sum_{j=1}^{N}R_{S}(i,j)=\sum_{j=1}^{N}\sum_{k=1}^{l}R_{S_{k}}(i,j)=\sum_{k=1}^{l}\sum_{j=1}^{N}R_{S_{k}}(i,j)\\ \;=\sum_{i\in {\tilde{S}}_{k},k=1}^{l}\left(n-1\right)=C_{i}(n-1) \end{array}$$ (A-12)

Therefore,

$$C_{i}=\frac{1}{n-1}\sum_{j=1}^{N}R_{S}(i,j)$$ (A-13)

According to the assumption described with regard to **Property 2**, namely, that the geospatial information service system has been running for a sufficiently long time, we know that *l* is also sufficiently large and that $\underset{S_{k}\in S}{P}(i\in S_{\;k})≅\underset{S_{k}\in S}{\bar{P}}(S_{\;k}\in {\tilde{S}}_{i}))$. Therefore, we find that

$$\underset{S_{k}\in S}{P}(i\in S_{\;k})≅\underset{S_{k}\in S}{\bar{P}}(S_{\;k}\in {\tilde{S}}_{i}))=C_{i}/l=\frac{1}{(n-1)l}\sum_{j=1}^{N}R_{S}(i,j)$$ (A-14)

Thus,

$$\begin{array}{l} P(f_{i})=\frac{1}{n}\underset{S_{k}\in S}{P}(i\in S_{k})≅\frac{1}{n(n-1)l}\sum_{j=1}^{N}R_{S}(i,j)\\ \;=\frac{1}{(n-1)M}\sum_{j=1}^{N}R_{S}(i,j)=\frac{1}{K_{S}}\sum_{j=1}^{N}R_{S}(i,j) \end{array}$$ (A-15)

Eq (A–15) shows that the access probability of one small file is proportional to the sum of the elements in the corresponding row (column) of *R*_*S*_.

### Property 4

According to Eq (A–10), we can also write the following:

$$P_{S}(f_{j}|f_{i})=\frac{\underset{S_{k}\in S}{P}(j\in S_{k}|i\in S_{k})}{n-1}=\frac{\underset{S_{k}\in S}{P}(j\in S_{k},i\in S_{k})}{\left(n-1)\underset{S_{k}\in S}{P}(i\in S_{\;k}\right)}$$ (A-16)

As in the proof of **Property 3**, because of the large value of *l*, we have $\underset{S_{k}\in S}{P}(i\in S_{\;k},j\in S_{k})≅\underset{S_{k}\in S}{\bar{P}}(i\in S_{\;k},j\in S_{k})$. Using the same analysis presented for Eq (A–11), we can write the following:

$$\underset{S_{k}\in S}{\bar{P}}(i\in S_{\;k},j\in S_{k})=\frac{R_{S}(i,j)}{l}$$ (A-17)

In combination with Eqs (A–14) and (A–17), Eq (A–16) can be rewritten as follows:

$$\begin{array}{l} P_{S}(f_{j}|f_{i})=\frac{\underset{S_{k}\in S}{P}(j\in S_{k},i\in S_{k})}{\left(n-1)\underset{S_{k}\in S}{P}(i\in S_{\;k}\right)}=\frac{R_{S}(i,j)}{l}\frac{1}{\left(n-1)\underset{S_{k}\in S}{P}(i\in S_{\;k}\right)}\\ \;=\frac{R_{S}(i,j)}{(n-1)l}\frac{(n-1)l}{\sum_{j=1}^{N}R_{S}(i,j)}=\frac{R_{S}(i,j)}{\sum_{j=1}^{N}R_{S}(i,j)}\\ \;=K_{S}R_{S}(i,j)/P(f_{i}) \end{array}$$ (A-18)
